# Role of the C-terminal modules of Klebsiella phage KP32 receptor-binding protein gp38 in protein and phage functionality

**DOI:** 10.1371/journal.ppat.1014106

**Published:** 2026-04-06

**Authors:** Agnieszka Latka, Dorien Dams, Lennert Scholiers, Aleksandra Otwinowska, Sebastian Olejniczak, Zuzanna Drulis-Kawa, Yves Briers

**Affiliations:** 1 Department of Biotechnology, Ghent University, Ghent, Belgium; 2 Department of Pathogen Biology and Immunology, University of Wroclaw, Wroclaw, Poland; Pirbright Institute, UNITED KINGDOM OF GREAT BRITAIN AND NORTHERN IRELAND

## Abstract

Despite significant progress in understanding phage biology and their clinical applications, the specificity of phages remains only poorly understood and a matter of empirical testing. Phage receptor-binding proteins (RBPs), which mediate the initial contact with bacterial cells and govern host recognition, possess a modular architecture. The N-terminal domains primarily serve a structural role, facilitating the attachment of the RBP (or RBP complex) to the virion. In contrast, the function of the C-terminal modules, and their interplay with the central enzymatic domain in impacting RBP and phage specificity, remains underexplored. This study investigates the receptor-binding protein KP32gp38 of Klebsiella phage KP32, which contains an unusual C-terminal combination of a carbohydrate-binding module (CBM) and a lectin-like (LEC) domain, two elements that are typically found separately rather than in tandem. We dissected the roles of these modules in trimerization, substrate binding, and specificity at both the protein and phage level. By deletions, fusions, and exchanges of the modules through both protein and phage engineering, we examined the impact of the domains on the specificity of the RBP and the host range of the phage. Protein fusions with GFP were tested for their ability to bind the bacterial capsule. To verify the influence of the domains on RBP trimerization, different variants were analysed with SEC – MALLS. The results revealed that the LEC domain is essential for trimerization, whereas the CBM domain is crucial for enzymatic activity and capsule binding. Engineered phages lacking these domains confirmed the necessity of both CBM and LEC for full functionality. This work underscores the versatility and evolutionary adaptation of CBM and LEC folds in phage RBPs, providing valuable insights into phage specificity mechanisms. Our findings offer a blueprint for understanding the molecular determinants of phage-host interactions, crucial for advancing phage-based antibacterial therapies.

## Author summary

Phages are viruses that attack bacteria, and they are being studied as possible alternatives to antibiotics, which are losing their effectiveness. But one big question remains: how do phages know which bacteria to infect? In our work, we looked at a protein on the tail of a phage that helps it recognize its target. This protein has two parts that usually appear separately in nature, but here they are joined together. We changed these parts in different ways to see what would happen. We found that one part is needed to keep the protein’s shape, while the other part is needed to stick to the bacteria. Both are essential for the phage to work. Understanding this gives us clues about how phages adapt to their hosts and could help us design phages that target specific bacteria. This is an important step toward creating new treatments for infections when antibiotics fail.

## Introduction

Bacteriophages are viruses with a high specificity to their bacterial host. This specificity is an evolutionary dynamic trait defined by the concerted action of phage receptor-binding proteins (RBPs), phage defence systems, and the capacity of the phage to hijack the bacterial metabolism to produce progeny phage [1]. Recent advances in AI tools that aim to predict host specificity at the strain level highlight the significance of RBPs as the primary determinant for phage specificity [2,3]. Phage RBPs can exhibit either a tail fiber (TF) or tailspike (TSP) morphology. TFs are long fibrous structures, often bent in the middle, binding a bacterial receptor [4], whereas TSPs are shorter, thicker, and often equipped with enzymatic activity for carbohydrate degradation or modification [5]. While protruding loops in the most distal end have been identified as essential determinants for TF receptor binding and specificity [6–9], the specificity factors of TSPs remain enigmatic [10,11].

In the case of *K. pneumoniae,* many phage TSPs target a specific type of capsular polysaccharides [12–16]. The latter serves as a protective layer against harsh environmental conditions and the immunological responses of infected individuals. At least 79 capsular types (K-types) and 186 capsule genotypes (KL types) have been identified in *Klebsiella* spp. [17–19]. Correspondingly, Klebsiella phages display an equally high diversity of TSPs, which often share little to no amino acid sequence identity with each other. These TSPs degrade capsular polysaccharides to allow phages to gain access to the deeper layers and proceed with DNA injection.

**Fig 1:**
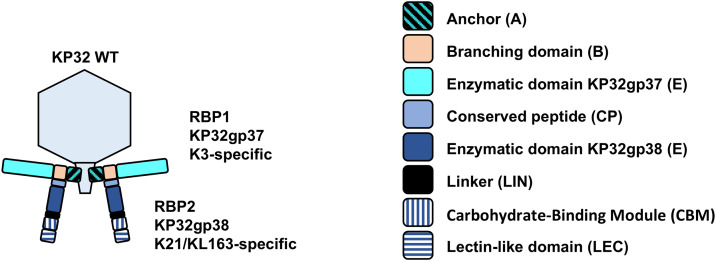
Model of the dual RBP system of the KP32 phage virion: RBP1 anchors the full RBP system to the virion via its anchor domain (A) and provides a branching domain (B) for RBP2 attachment through its conserved peptide (CP). RBP1 has a central enzymatic domain (E) targeting the K3 capsule type. RBP2 has an enzymatic domain (E) specific to K21/KL163 capsule types, connected via a linker (L) to an unusual C-terminal tandem of a carbohydrate-binding module (CBM) and a lectin-like domain (LEC), classified as a Class 1, Group F double-domain depolymerase architecture. This configuration, two consecutive carbohydrate-interacting domains, is rarely observed among phage tailspike proteins and represents a key focus of this study.

Despite a high sequence diversity impeding the understanding of capsular specificity, TSPs share a highly similar β-helical shape responsible for the enzymatic activity [20,21]. Additionally, conserved structural domains can be distinguished depending on whether the phage particle is equipped with a single RBP or multiple RBPs [22], as initially described for Escherichia and Salmonella phages [23]. Klebsiella phage KP32 belonging to the *Przondoviruses* has been studied as a model phage with two types of receptor-binding proteins (RBPs) (Fig 1). RBP1 (KP32gp37) recognizes the K3 capsular type, while RBP2 (KP32gp38) recognizes K21 and KL163 [14]. RBP1 is composed of two structural domains, *i.e.*, an anchor (A) and a branching domain (B), followed by an enzymatic domain (E) equipped with a C-terminal chaperone that is autoproteolytically cleaved off [22]. The anchor domain, responsible for attachment of the RBP system to the phage tail, is highly conserved among *Przondoviruses*. The branching domain provides an attachment site for RBP2 that attaches via a conserved peptide (CP) in its N-terminus. RBP2 (KP32gp38) consists of a conserved peptide (CP), an enzymatic domain (E), a linker (LIN), and an extraordinary C-terminal tandem of a carbohydrate-binding module (CBM) and a lectin-like domain (LEC). This tandem is now classified as a Class 1, Group F “double C-domain” depolymerase architecture, according to the recently proposed structural taxonomy of phage depolymerases [24]. Class 1, Group F depolymerases are characterized by two consecutive carbohydrate-interacting domains at the C-terminus, a configuration rarely observed in tailspike proteins. While CBM and LEC domains are individually common in carbohydrate-active proteins, their coexistence within a single tailspike protein is highly unusual and, to our knowledge, has not been experimentally dissected before. This rare pairing raises questions about their functional interplay and necessity.

CBMs are non-catalytic parts of larger carbohydrate-active enzymes. While some lectins are classified as CBMs, the feature distinguishing lectins from CBMs is their ability to cause agglutination of carbohydrate-containing molecules. Lectins tend to form dimers, trimers, or tetramers and possess multiple binding sites [25]. Squeglia et al. (2020) proposed earlier that the KP32gp38_CBM domain brings the enzymatic domain into close contact with the substrate, improving enzymatic degradation. For KP32gp38_LEC, both recognition and efficient binding to CPS, as well as securing RBP trimerization, were proposed. Putative polysaccharide-binding sites were predicted in both domains [26]. Since the coexistence of both domains in a single TSP was previously not yet described, the goal of this study was to unravel the impact of the distinct C-terminal domains on RBP specificity, protein multimerization, and phage infectivity through the investigation of truncated and chimeric variants of KP32gp38 at both protein and phage levels. Although the individual CBM or LEC domains have been previously predicted and their potential functions proposed, experimental verification remains limited. To our knowledge, the functional dissection of the C-terminal domains of phage RBPs with depolymerising activity, both at the protein level and within the context of the phage particle, has not yet been reported. In this study, we employed a highly systematic approach to generate 52 truncated and chimeric variants of KP32gp38 RBP. Additionally, six distinct phages were engineered to evaluate the role of the C-terminal CBM and LEC domains in determining RBP specificity and overall phage functionality.

## Results

### RBP2 (KP32gp38) lacking the LEC domain retains enzymatic activity with a conserved specificity, but deletion of CBM or CBM-LEC results in a complete activity loss

To understand the function of the individual domains, a systematic collection of fourteen truncated RBP2 variants missing one or more domains was constructed and produced, along with the original wild-type (WT) as a control (Table 1). The different protein variants were subsequently analyzed for their enzymatic activity through a spot assay on bacterial lawns using strains targeted by the WT RBP2 (strain Kp358 and Kp968 with the K21 and the KL163 capsular type, respectively). The activity was evaluated with a two-fold protein dilution series and observed as a semi-transparent halo zone, resulting from capsule degradation. Finally, the minimum halo-forming concentration (MHC) was determined as a semi-quantitative measure of activity.

**Table 1:** Enzymatic activity of truncated variants and individual domain(s) of RBP2 (KP32gp38). An overview of the WT and its truncated derivatives constructed and analyzed in this work is visualized in the scheme. The corresponding name and the delineated amino acids referring to the WT RBP2 are shown along with the present and deleted domains (in the N-to-C direction). CP = conserved peptide; E = enzymatic domain; LIN = linker between E and CBM; CBM = carbohydrate-binding module; LEC = lectin-like domain. The colour scheme and abbreviations correspond to the RBP2 model presented in Fig 1. The enzymatic activity of all variants was tested on the *K. pneumoniae* strains with capsular types recognized by the WT RBP2: Kp358 (K21) and Kp968 (KL163). K-type indicates the presence of enzymatic activity, whereas NO indicates the absence of any observed activity on those strains. 3D structures predicted using AlphaFold 3 [27] can be found in Fig A in S1 File.

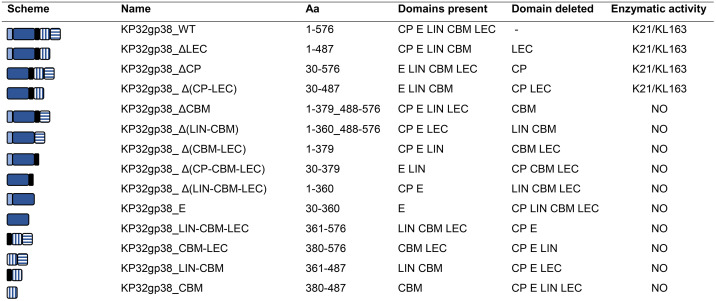

The truncated variant KP32gp38_ΔLEC, deprived of its ultimate C-terminal domain (LEC), showed a 1,250–5,000-fold activity reduction compared to the WT RBP2 (MHC of 78.125 nM and 312.5 nM for K21 and KL163, respectively, compared to 0.0625 nM, Table A in S1 File). Assuming that LEC narrows the specificity spectrum of RBP2, its removal could potentially broaden the RBP2 spectrum. Therefore KP32gp38_ΔLEC was further screened on a broad *Klebsiella* capsular type collection comprising 77 different types (Table B in S1 File). Among 92 strains tested, only those classified as K21 (Kp358, Kp45, Kp52.225 CIP) or KL163 (Kp968) capsular types were susceptible to KP32gp38_ΔLEC, refuting the hypothesis of spectrum extension. We, therefore, concluded that the LEC domain is not responsible for capsular type specificity. Instead, its presence may correlate with increased enzymatic activity, potentially through improved protein folding or by contributing to substrate binding, for example, by facilitating a more favorable orientation toward the substrate. Additionally, because the catalytic pocket is located at the interface between monomers, multimerization is required for enzymatic activity. Two other truncated proteins, specifically KP32gp38_ΔCP and KP32gp38_Δ(CP-LEC), also retained their activity and specificity towards K21/KL163. This was not unexpected since the CP domain located at N-terminus was proven to serve as an anchoring structure [26].

Other truncated variants with deletion of both C-terminal domains or deletion of the CBM domain alone, both with or without linker (*i.e.*, KP32gp38_ΔCBM, KP32gp38_Δ(CBM-LEC), KP32gp38_Δ(LIN-CBM), KP32gp38_ Δ(CP-CBM-LEC)) were expressed as soluble proteins lacking enzymatic activity on K21/KL163 strains. In addition, individual domains or a combination of CBM, LEC and/or linker resulted in well-overexpressed proteins in the soluble fraction without any enzymatic activity against capsular types from the tested collection (Tables 1 and B in S1 File).

### CBM and LEC do not transfer specificity to other RBPs

**Fig 2:**
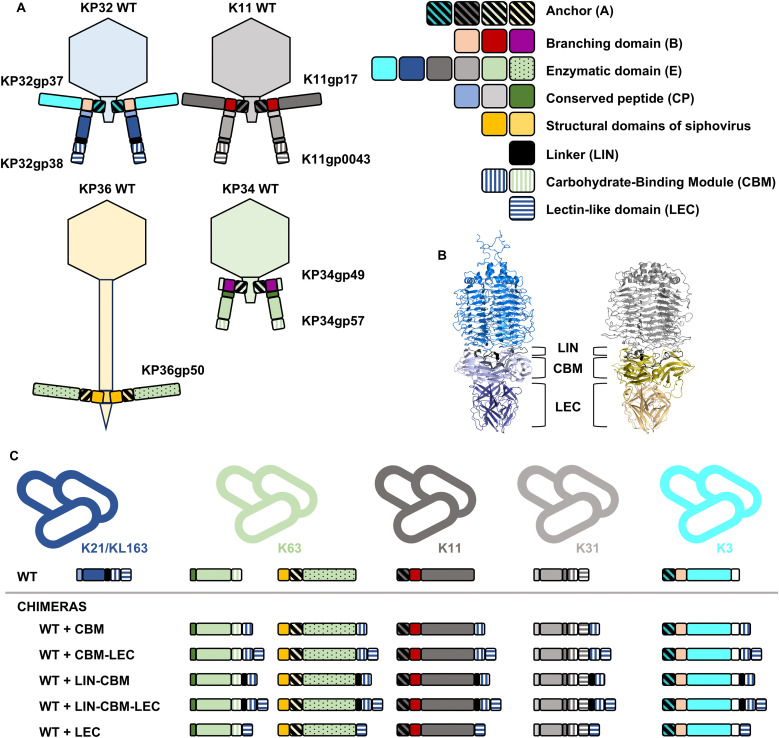
Klebsiella phages and their RBPs selected for protein fusions along with the chimeric protein specificity. (A) Models of RBP systems of Klebsiella phages with podovirus morphology (KP32, K11, and KP34) and siphovirus morphology (KP36). (B) 3D structure of KP32gp38 (KP32gp38_CP-E (marine), KP32gp38_LIN (black), KP32gp38_CBM (light blue), KP32gp38_LEC (deep blue)) and K11gp0043 (K11gp0043_CP-E (light grey), K11gp0043_LIN (black), K11gp0043_CBM (olive), K11gp0043_LEC (light orange)) modelled with AlphaFold 3 [27]. (C) Protein fusions along with their capsular type (K-type) specificity compared to the WT RBPs. The 3D structures predicted using AlphaFold 3 [27] can be found in Figs B-C in S1 File.

To further investigate whether the CBM or LEC domain impacts specificity towards certain capsular types, two approaches were used. First, other RBPs with depolymerase activity were directly fused with the RBP2 C-terminal domains (*i.e.*, CBM, LIN-CBM, CBM-LEC, LIN-CBM-LEC, LEC) to analyze whether these domains can bring any additional specificity to existing enzymatic proteins. RBP1 (KP32gp37) from phage KP32, RBPs from the closely related *Przondovirus* phage K11, as well as *Drulisvirus* phage KP34 RBP and *Webervirus* KP36 RBP of siphovirus morphology were tested (Fig 2). The KP34gp57, located at the position of RBP2 in the virion and equipped with a predicted CBM domain, had the same specificity towards the K63 capsular type, as KP36gp50 originating from the selected siphovirus. All 25 chimeras designed in the first approach (Fig 2 and Table C in S1 File) were recombinantly produced and screened on the full *Klebsiella* capsular type collection (Table D in S1 File). All chimeric proteins were active and specific to the capsular type corresponding to the N-terminus of the original RBP. There was no specificity switch or extension of chimeric enzymes.

In a second approach, analogous C-terminal CBM and LEC domains of RBP2 of the closely related *Przondovirus* phage K11 (K11gp0043) were exchanged with those of phage KP32 RBP2 to investigate whether the specificity of these chimers can be switched or extended depending on the present C-terminal domains. The predicted 3D structure and amino acid sequences of phage K11 RBP2 were compared to the crystal structure of phage KP32 RBP2 to delineate the same domains (*i.e.*, CP, E, LIN, CBM, LEC). Six chimeric proteins were prepared and screened on K21 (Kp358), KL163 (Kp968), and K31 (Kp52.231 CIP) recognized by the parental proteins (Table 2). Overexpression was detected on SDS-PAGE for KP32gp38_CP-E_K11gp0043_LIN-CBM-LEC and KP32gp38_CP-E-LIN_K11gp0043_CBM-LEC, but enzymatic activity was only observed for the former one at a high concentration (25 µM). Moreover, this protein was active on K21 but not consistently on KL163 and it was not active on K31. Its specificity partially corresponds to KP32gp38 specificity thus the presence of the C-terminal CBM and LEC domains derived from K11gp0043 did not impact the specificity of KP32gp38_CP-E. Notably, the K11gp0043 CBM and LEC domains allowed for overexpression of the chimeric protein in a soluble form. However, the mirrored protein K11gp0043_CP-E_KP32gp38_LIN-CBM-LEC and hybrid CBM/LEC combinations could not be successfully expressed as soluble proteins. The reduced or lack of solubility for these chimeras could be in part explained by the two-amino acid junctions resulting from the assembly process as shown for the parental proteins (Table E in S1 File), highlighting the relevance of orientation and concerted action of all domains for proper folding and functionality.

**Table 2:** Enzymatic activity of chimeric variants obtained by the fusion of KP32gp38 and K11gp0043 domains. A blue colour indicates a KP32 origin, whereas grey indicates a K11 origin. LIN = linker between enzymatic domain and CBM; CBM = carbohydrate-binding module; LEC = lectin-like domain. NO = protein expression was observed on SDS-PAGE, but no halo was observed; nd = protein expression was not detected on SDS-PAGE, and a halo was not formed on tested lawns (K31, K21, KL163). The colour scheme and abbreviations correspond to the models presented in Fig 2. The green triangle indicates where a two-amino acid position marker was added. * inconsistent results for KL163. The 3D structures predicted using AlphaFold 3 [27] can be found in Fig D in S1 File.

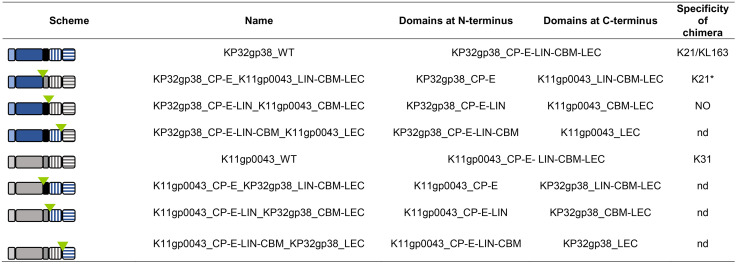

### The full-length RBP2 binds to capsulated bacteria in contrast to the C-terminal modules alone

Next, the binding potential to the bacterial surface was tested for KP32gp38 separate domains. Green fluorescent protein (GFP) was fused with RBP2 derivatives and then co-incubated with bacteria. Unbound proteins were removed by washing and the GFP-labelled cells were detected fluorometrically. GFP fusions to full-length KP32gp38 showed significant binding to the expected capsular type (KL163) and not to the non-target capsular types (K3, K63) but only if the protein was enzymatically inactivated as a triple mutant (E170Q-E239Q-D241N; < 0.002% residual activity; [26]) (Fig E in S1 File). The absence of binding for the WT GFP fusion can be explained by the high enzymatic activity of this protein, which rapidly digests the capsule on the bacterial surface.

**Fig 3:**
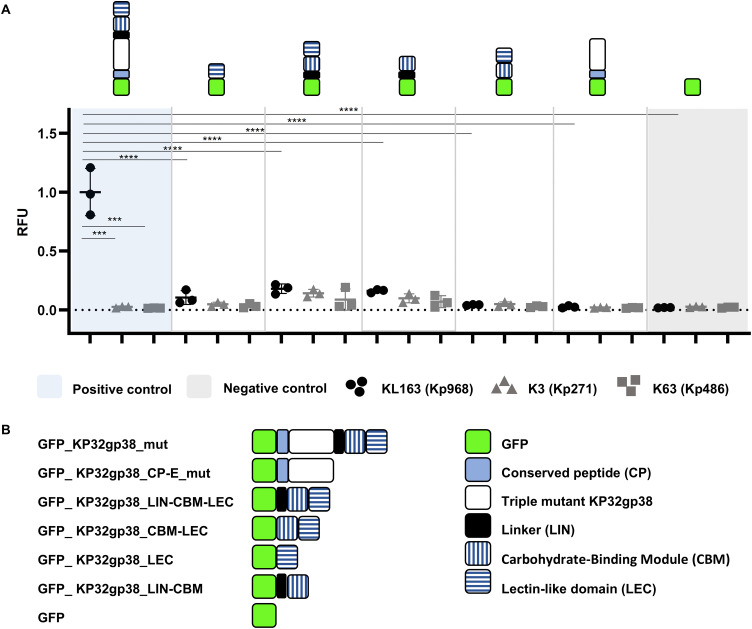
Fluorescence-based binding assay – C-terminal modules alone are not responsible for binding to the capsular receptor. (A) KP32gp38 modules were fused with a GFP tag on the N-terminus for binding with different *K. pneumoniae* strains of capsular type KL163 (Kp968 – circle), K3 (Kp271 – triangle) and K63 (Kp486 – square). The KL163 capsule degraded by the WT enzyme is indicated with black symbols, and non-targeted types (K3 and K63) are marked with grey symbols. Results for the GFP-tagged modules, GFP alone, and the GFP_KP32gp38_mut are presented on a white, grey, and blue background, respectively. Constructs tested are depicted on top of the respective results. Stars indicate statistically significant differences: *** for p < 0.001, **** for p < 0.0001 (Ordinary one-way ANOVA, GraphPad Prism 9.0.0). **(B)** Overview of the different chimeric fusions. The 3D structures predicted using AlphaFold 3 [27] can be found in Figs F-G in S1 File.

Upon assay validation, different chimeric fusions with the GFP tag at the N-terminus and a variable set of domains from the KP32gp38 triple mutant were produced and analyzed against the same panel of strains (Fig 3). We found that only the full-length protein can significantly (p < 0.0001) bind to the bacteria of the targeted capsule polysaccharide. Protein lacking the CBM-LEC domains, as well as the CBM or LEC domains alone, in tandem, or CP-E alone did not bind to the bacterial cell surface (p-value ranging from 0.2581 to 0.9999).

**Fig 4:**
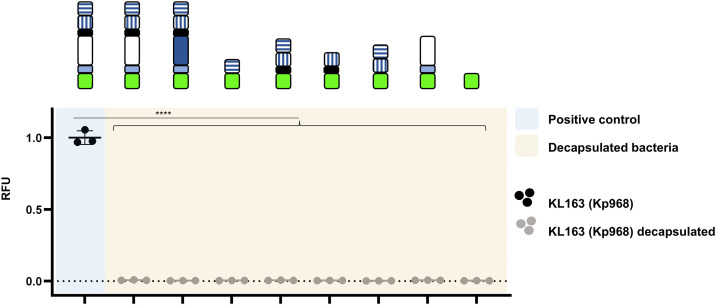
Fluorescence-based binding assay with decapsulated strains – C-terminal modules are not responsible for binding to the secondary receptor. Different modules of KP32gp38 were fused with GFP-tag on N-terminus and tested with *K. pneumoniae* strains of capsular type KL163 (Kp968 – circle). The strain with a capsular type KL163 to which the positive control (GFP_KP32gp38_mut) binds is indicated with black symbols. Decapsulated KL163 cells, to which the positive control does not bind, are marked with grey symbols. Results for positive control are presented on a blue background, decapsulated bacteria are depicted on a yellow background. Constructs tested are depicted on top of the respective results. Stars indicated statistically significant differences, tested between each variable: **** for p < 0.0001 (Ordinary one-way ANOVA, GraphPad Prism 9.0.0).

We further investigated whether KP32gp38 and its C-terminal domains could potentially bind to a non-capsule-related (secondary) receptor located beneath the capsule. Therefore, the capsule was removed from Kp968 cells (KL163 type) through enzymatic degradation by the KP32gp38 WT depolymerase prior to the binding assay (Fig E in S1 File). It turned out that none of the constructs including the GFP_KP32gp38_mut could bind to decapsulated bacteria (Fig 4). The latter indicates successful capsule removal, the necessary presence of intact capsule for KP32gp38 binding, and the lack of secondary receptors targeted by CBM or LEC. These observations are in line with earlier reports that capsule loss results in protection against CPS targeting phages [15].

### LEC is sufficient to facilitate proper protein trimerization, while CBM assists enzymatic activity

The CBM and LEC domains neither impacted the specificity nor showed autonomous capsule binding capacity; however, they are necessary to enable capsule binding for the full-length protein. It was earlier found that the catalytic pocket of KP32gp38 is located at the interface of independent monomers (intersubunit catalytic pocket), highlighting the necessity of trimerization for enzymatic activity [26]. Therefore, we investigated the role of the CBM and LEC domains in trimerization. Selected truncated variants, specifically C-terminally truncated proteins KP32gp38_ΔLEC and KP32gp38_CP-E, as well as the C-terminal domains KP32gp38_LIN-CBM-LEC, KP32gp38_CBM, and KP32gp38_LEC, were subjected to size exclusion chromatography (SEC) - Multi-Angle Laser Light Scattering (MALLS) analysis to assess the molecular weight (MW) of their oligomeric state (Table 3). Full-length KP32gp38_WT naturally adopts a trimeric structure, composed of three identical monomers. Deletion of the LEC domain led to the mixture of monomers (~71%) and some dimers (~10%) for KP32gp38_ΔLEC in protein suspension. KP32gp38_ΔLEC was the only truncated variant possessing enzymatic activity but with an ~ 1,000 fold activity drop compared to the WT (Table 3), which can be explained by the disrupted trimerization. Further protein truncation resulted in more complex protein oligomerization states, with about half of the KP32gp38_CP-E proteins forming monomers and the remaining part undergoing different states of multimerization (dimers, trimers, pentamers, and hexamers). CBM and LEC domains, individually present in the solution, tend to form large multimeric structures or aggregates, while prepared in a combined version (KP32gp38_LIN-CBM-LEC) again adopted a trimeric state, similar to the WT protein. Finally, the oligomeric state of GFP_KP32gp38_LIN-CBM-LEC was also evaluated to investigate the impact of an N-terminal GFP-tag on the proper protein trimerization and it was found to form a trimer. Therefore, misfolding cannot explain the lack of binding to the capsulated cells observed in the previous experiments. The presence of CBM or LIN-CBM was not required for protein trimerization, but the enzymatic activity was lost without these domains. Thus, the LEC domain was sufficient to facilitate proper protein trimerization but CBM was essential for enzymatic activity. Fusion of the KP32gp38 enzymatic domain with non-cognate CBM-LEC or LIN-CBM-LEC domains from phage K11 also resulted in trimeric proteins, however, with significantly reduced enzymatic activity (0.0005% of WT activity), confirming the role of a cognate CBM for enzymatic activity. Enzymatic activity of trimeric KP32gp38_CP-E-LIN_K11gp0043_CBM-LEC was not detected, even further emphasizing the importance of proper CBM orientation in the substrate fitting. In conclusion, the ultimate C-terminal LEC domain facilitates the oligomerization of KP32gp38 into a trimeric structure. This conformation is important for enzymatic activity due to the inter-subunit catalytic pocket location and for effective binding to the bacterial CPS. However, trimerization does not suffice and the presence of CBM is required to obtain full enzymatic activity. The CBM may assist in substrate binding/positioning.

**Table 3:** Summary of oligomeric state and enzymatic activity of selected truncated variants of KP32gp38. The colour scheme and abbreviations correspond to the models presented in Tables 1 and 2. nm – not measured, large particles were flowing from the column. The 3D structures predicted using AlphaFold 3 [27] can be found in Figs A, D, F and G in S1 File.

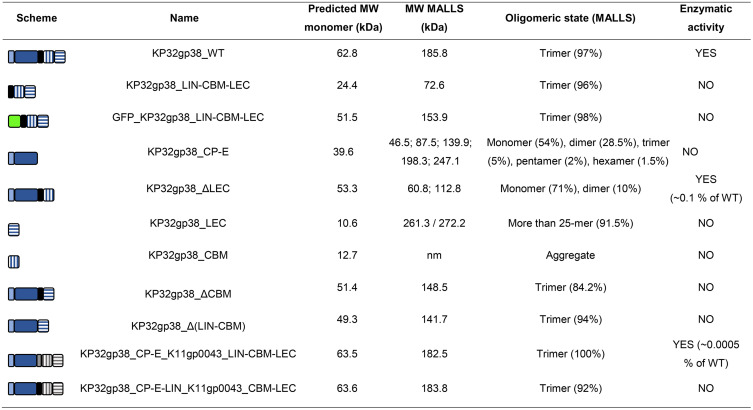

### The presence of RBP2 is not essential for virion assembly and infection

**Fig 5:**
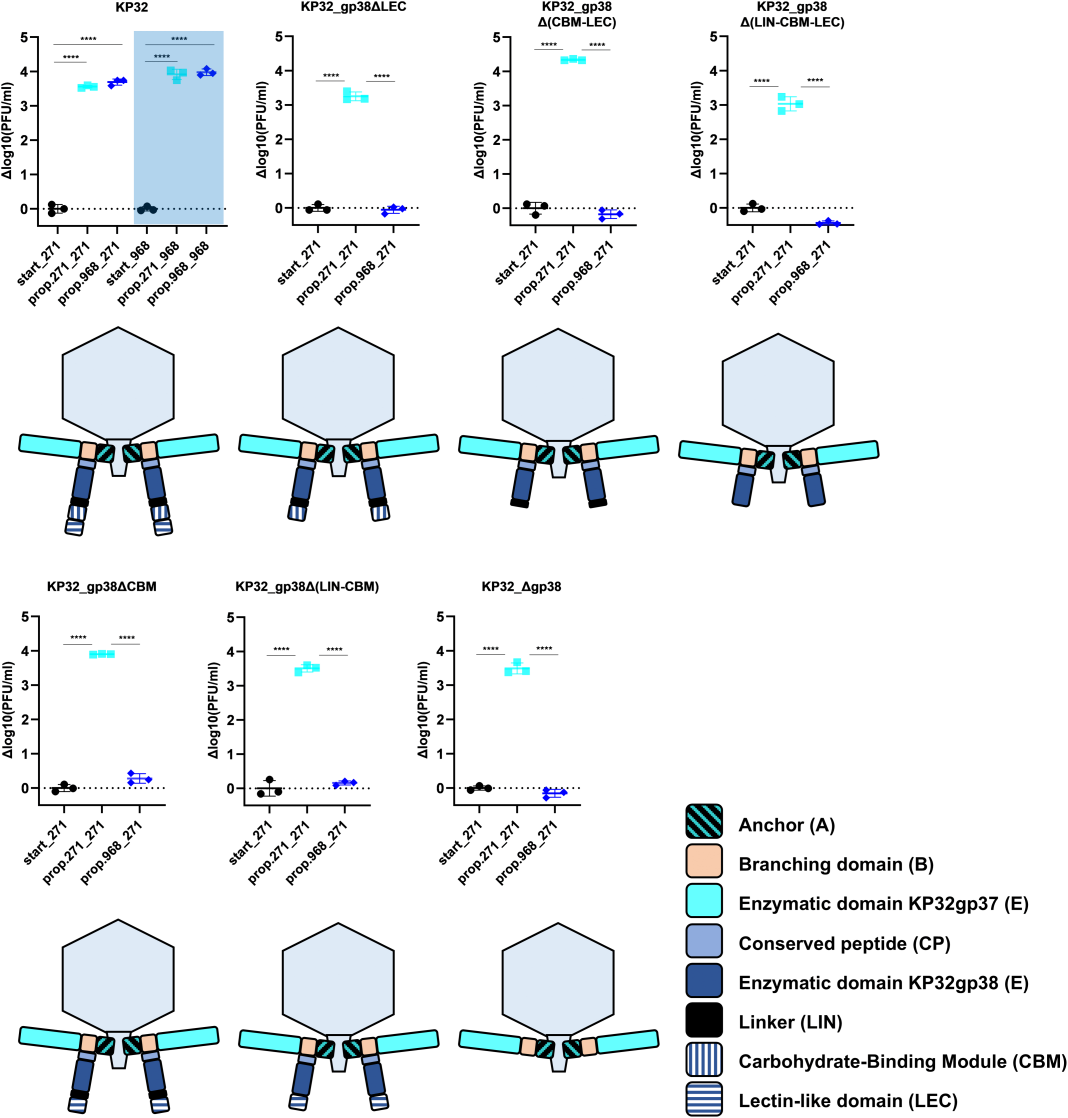
Engineered phage propagation on hosts for RBP1 and RBP2. The results are presented as log10 changes in phage titer (Δlog10(PFU/mL) after propagation on each host compared to the start phage titer. The start phage titers are depicted as black circles; propagation on Kp271 (K3) – RBP1 host as cyan squares; propagation on Kp968 (KL163) – RBP2 host as dark blue diamonds. “start” – Δlog10(PFU/mL) of the phage suspension before propagation, “prop.” – Δlog10(PFU/mL) of the phage suspension after propagation. The first bacterial strain indicates on which propagation cycles were done, whereas the second bacterial strain indicates on which spotting was done. Phages plated on a Kp271 (K3) lawn are presented on a white background, and those on Kp968 (KL163) on a blue background (only for KP32, as engineered phages were unable to propagate on Kp968 and did not form plaques on this strain). With **** statistically significant differences, for p < 0.0001, are indicated (Ordinary one-way ANOVA, GraphPad Prism 9.0.0).

Finally, we evaluated how the specified roles of the C-terminal domains at the RBP level can be translated to their functionality at the phage level. Different variants of phage KP32 with a truncated RBP2, deprived of at least one of the C-terminal domains, were engineered and rebooted to infective phage particles. In all cases, RBP1 remained intact (Fig 5). Subsequently, all engineered phages and the WT phage KP32 underwent three propagation cycles on two hosts each: *K. pneumoniae* Kp271 (K3) recognized by RBP1, and *K. pneumoniae* Kp968 (KL163) recognized by RBP2. After three rounds of propagation, the phage titer was determined on both hosts (for RBP1 and RBP2). The increase in phage titer was expressed relative to the titer of the starting, non-propagated phage suspension to quantify effective propagation (Fig 5). Phage KP32 was able to propagate equally on both hosts, reaching an increase of 3.6 and 3.7 Δlog10 PFU/mL on *K. pneumoniae* K3 and KL163, respectively. All engineered phages with a truncated RBP2 could effectively propagate on the host targeted by RBP1 (3.0 - 4.3 Δlog10 PFU/mL), also when completely deprived of RBP2 (3.5 Δlog10 PFU/mL) when propagated on the host for RBP1. In contrast, none of the engineered phages with a truncated or deleted RBP2 was able to propagate on the RBP2 host (Kp968). These observations at the phage level align with the findings at the protein level. A non-trimeric RBP2 is associated with a lack of propagation by the corresponding phage, either due to a lack of assembly in the phage particle or a lack of RBP2 enzymatic activity. Phages equipped with KP32gp38ΔLEC of reduced enzymatic activity could not effectively propagate.

## Discussion

### Phage KP32 is studied as a model phage for its dual RBP system with an unusual CBM-LEC tandem in RBP2

Inspired by earlier work on Escherichia phages G7C [28], CBA120 [29], K1-5 [23], and other K1-specific phages [30], Salmonella phages SP6 [31,32] and P22 [33,34], and Shigella phage Sf6 [33,35], Klebsiella phage KP32 has evolved in the past years to a well-studied Klebsiella phage with a dual RBP system. The depolymerase activity of both RBPs has been extensively characterized at the biochemical level, and their antibacterial potential as antivirulence compounds, sensitizing cells for serum and phagocytosis, was evaluated [14], as well as the crystal structure of RBP2 (KP32gp38) determined [26]. The modular build-up of the dual RBP system was revealed in detail and experimentally confirmed through the creation of numerous chimeric proteins at the protein and phage level, mimicking the natural horizontal transfer process [22,36]. This blueprint of RBP modularity is universal and can be expanded to phages with more complex RBP systems [37].

Both RBPs of phage KP32 have (an) additional C-terminal module(s). In the case of RBP1 (KP32gp37), this domain was shown to be an autoproteolytically removed chaperone that is necessary for folding and/or trimerization [14,22]. In contrast, RBP2 (KP32gp38) possesses two C-terminal carbohydrate-interacting modules that are not cleaved off. The crystal structure of KP32gp38 provided the first experimental evidence of a CBM–LEC tandem in an RBP [26], now recognized as a hallmark of Class 1, Group F depolymerases [24]. This structural category is defined by two consecutive carbohydrate-interacting domains at the C-terminus, a configuration rarely observed among tailspike proteins. Our study delivers the first functional dissection of this architecture, demonstrating that the tandem is not merely structural but plays distinct roles: LEC drives trimerization, while CBM assists enzymatic activity and substrate positioning.

Homology searches indicated that this architecture is sporadically distributed among Klebsiella and related phages. The CBM–LEC tandem was found in Klebsiella phages PMBT70 (XDJ01157.1), K5 (YP_009198669.1), K64-1 (YP_010843553.1), Kp_Pokalde_001 (QWT56644.1) and Stenotrophomonas phage BUCTxx100 (WPH68638.1). For four other proteins from Klebsiella phages Saitama (WEU80486.1), K11 (YP_002003831.1), pKP-BM327-1.1 (UZN24474.1) and vB_KqM-Westerburg (CAD5240880.1) the amino acid sequence similarity covers the CBM but not the LEC domain. In contrast, there are numerous reports on separate CBM and lectin-like domains in the C-terminus of phage RBPs. CBM-like domains were found in RBPs of Escherichia phages G7C (a tandem of two CBM-like domains in gp63.1) and CBA120 (TSP4, orf213, gp165), as well as in Klebsiella phages RAD2 (gp02) and GH-K3 (gp32) [15,16,28,29]. Two CBMs of gp63.1 of phage G7C were predicted to form a valley important for LPS binding, together with the esterase domain of another monomer [28]. CBMs of CBA120, RAD2, and GH-K3 phage RBPs were proposed to participate in binding to cell surface oligo- and polysaccharides. The lectin-like fold alone was previously mentioned for the RBP of R-pyocins and *Acinetobacter baumannii* phage AP22, where its involvement in LPS binding was suggested [8,38,39]. Additionally, the presence of a domain with a putative oligosaccharide binding function was described for Pseudomonas phage LKA, Acinetobacter phage AP22, and Shigella phage Sf6 [40]. The authors hypothesized that these domains evolved to bind to oligo- and polysaccharides as well as stabilize the trimers formed by RBPs [40].

### Trimerization of the enzymatic domain is not sufficient for activity

Phage RBPs naturally form trimers with six copies present in a virion [23]. While the β-helical fold of the enzymatic domain is universal among TSPs, more diversity in the location and shape of the active site exists. The catalytic pocket of the TSPs of Escherichia phage HK620 and Salmonella phage P22 is located in a single monomer (intra-subunit), whereas in KP32gp38 and the Shigella phage Sf6 TSP the pocket lies between the β-helix of two monomers (inter-subunit location) [26,33,35,41]. Some TSPs (*i.e.*, KP34gp57–8BKE, LKA1gp49–4RU4, phi297gp61–4RU5, AP22gp54 - 4Y9V, Det7 - 2V5I, 9NA - 3RIQ) possess an additional so-called insertion domain, which in the case of KP34gp57 complements the β-helical domain in the formation of an intra-subunit catalytic pocket [42]. A similar positioning of the catalytic cavity was predicted for LKA1gp49, where the insertion domain makes the substrate-binding groove of the β-helix deeper and participates in the formation of the active site [40]. This contrasts with other TSPs that lack such an insertion domain and instead feature a catalytic cleft located at the interface between two monomers of the trimer, forming an inter-subunit catalytic pocket. Additionally, in the case of KP34gp57, the authors showed that a functional monomeric ‘mini-enzyme’ variant of KP34gp57 could be created by C-terminal truncation of its CBM, which is responsible for the trimerization of KP34gp57. Trimerization appears thus essential for a TSP with an inter-subunit catalytic pocket as revealed for KP32gp38 but not for a TSP with an intra-subunit catalytic pocket. However, trimer formation does not suffice since the trimer KP32gp38_ΔCBM is not active, whereas the non-trimeric KP32gp38_ΔLEC retains a residual activity of 0.1%. These observations indicate that the presence of the CBM is also required for catalytic activity without being necessary for trimerization. This again contrasts with the ‘mini-enzyme’ derived from KP34gp57 whose trimmed C-terminal CBM domain was not required for activity but essential for trimerization. In addition, the exchange of the cognate CBM-LEC tandem of KP32gp38 by the non-cognate CBM-LEC tandem from phage K11gp0043 formed two soluble trimeric variants (KP32gp38_CP-E_K11gp0043_LIN-CBM-LEC and KP32gp38_CP-E-LIN_K11gp0043_CBM-LEC) with no or very limited (0.0005%) residual enzymatic activity. These observations underline again that trimerization does not suffice and that the cognate CBM is needed for proper activity.

Such variable contribution of a CBM domain to the overall activity has been reported before for other carbohydrate-active enzymes. Deletion of the CBM may lead to partial or complete loss of enzymatic activity due to impaired substrate binding [43], whereas other CBM-equipped enzymes showed no obvious reduction in catalytic activity upon CBM deletion [44,45]. Consistent with other CBMs and LECs [46], none of our constructs comprising merely CBM and/or LEC (KP32gp38_CBM; KP32gp38_LEC; KP32gp38_CBM-LEC) showed enzymatic activity.

### The LEC domain is responsible for trimerization

All our results support that the LEC domain is responsible and sufficient for KP32gp38 trimerization, which is also consistent with the multimerising ability assigned to lectins [25]. The individual LEC domain extensively multimerizes to more than 25-mers. Only, when a preceding domain is present, proper trimers are formed (KP32gp38_LIN-CBM-LEC, GFP_KP32gp38_LIN-CBM-LEC, KP32gp38_ΔCBM, and KP32gp38_Δ(LIN-CBM). Whereas the CBM of KP32gp38 is not required for trimerization and the individual CBM domain forms aggregates, the structurally similar CBM domain of KP34gp57 (DALI Z = 7.8, RMSD = 3.1 Å, sequence identity 6%; [42]) is crucial for CBM trimerization in KP34gp57. The KP32gp38_LEC and KP34gp57_CBM domains therefore appear to have equivalent functions. This also generally reflects the inconsistencies in the prediction and description of CBM or LEC domains throughout many studies and the challenges to differentiate CBM and LEC domains. For example, DALI searches for structural homologs of the CBM and LEC domain of KP32gp38 frequently yielded the same protein (e.g., TSPs from Escherichia phage G7C (PDB 4qnl), Klebsiella phage GH-K3, (PDBs 7vz3 and 7lzj), Cyanophage A-1(L) (PDB 8ke9) and Lactococcal phage 1358 (PDB 4l92).

### Capsule binding of RBP mediated through all domains with a prominent CBM role

The binding assays showed that RBP binding to the capsule only takes place when all domains are present (and the catalytic residues are mutated). Binding capacity could thus not be assigned to a single autonomous domain but rather results from the cooperative action of the enzymatic, CBM, and LEC domains. Indeed, besides residues in the catalytic pocket, polysaccharide binding sites were detected in the CBM and LEC domains [26]. The data suggests that CBM has a more prominent role in this multivalent binding process than the LEC domain, which has a more prominent role in trimerization. Generally, two CBM groups can be distinguished based on their affinity and mode of recognition: group I CBMs completely enclose their carbohydrate ligand and bind tightly, and group II CBMs bind a portion of the carbohydrate ligand and bind weakly [47]. Group II includes CBMs specific to polysaccharides, such as those in phage TSPs. Weak binding is in such cases compensated by multivalent interactions, coming from different domains, or occasionally from multiple binding sites on an individual CBM (*e.g.*, CBM6 has two binding sites). A similar multivalent interaction was proposed for phage G7C gp63.1, which is equipped with an O-antigen binding site in its esterase domain and a putative saccharide-binding site in the CBM-like domains 5 and 6. Thus a total of nine putative binding sites per trimer were likely evolved to function together, driving the enzyme and the phage particle through the LPS layer toward the host cell surface [28]. This approach also aligns with the notion that a too-tight binding would impair enzyme processivity [47]. Highly processive TSPs appear to be of utmost importance to penetrate a thick capsule layer to achieve successful infection of bacterial hosts.

### RBP1 of phage KP32 function independently from RBP2

We evaluated the impact of RBP2 truncation on the phage infectivity while keeping RBP1 intact. All phages with a truncated or deficient RBP2 lost the potential to infect the host targeted by RBP2. This is consistent with the lack of any relevant enzymatic activity and capsule binding capacity in all RBP2 truncations. Nevertheless, the infectivity of the host based on RBP1 was maintained, indicating that RBP1 can operate fully independently of RBP2. This is a relevant observation from the perspective of the signal cascade that occurs from irreversible receptor binding to genome ejection and cell entry. This cascade must be tightly controlled to avoid false infection events and unnecessary DNA spills, and different mechanisms for signal transmission have been described depending on RBPs’ nature [48]. In myovirus T4, recognition of the host receptor by the RBP induces a rotation of the baseplate protein gp10. This conformational change triggers a process of irreversible host binding [49]. A similar mechanism is suggested for *E. coli* podovirus G7C, which has also a branched complex of two RBPs (gp63.1-gp66). Gp66 is connected to the virion and provides an attachment site for gp63.1. Because the attachment site shows a structural similarity to the T4 gp10 protein, a similar signal transmission cascade via rotation or reorientation of the gp63.1-gp66 complex was suggested [28]. Also, the branching domain of RBP1 (KP32gp37) shows similarity to T4 gp10 [22], hinting at a similar mechanism. In addition, phages belonging to group C KP32viruses have a naturally truncated, branched RBP system where the RBP2 has only retained a small domain docking on the branching domain lacking enzymatic domain [22]. The latter suggested that physical interaction with RBP2, even when truncated and not functional in terms of host spectrum, is essential in the signal transmission mechanism. However, our current observations, specifically with the engineered phage KP32_Δgp38, refute this hypothesis, at least for phage KP32. The ability of KP32_Δgp38 – completely lacking RBP2 – to propagate on the RBP1 host indicates that rotation of the RBP complex is not required for signal transduction and a structural reorganization necessary to initiate the infection.

KP32gp38, as RBP2 could be compared to some podovirus RBPs forming a branching system. First, phage G7C gp63.1, which deacetylates the O-antigen in a processive manner, and second, Salmonella phage SP6 with two RBPs that both depolymerize the O-antigens. However, phage SP6 has an intermediate adaptor protein to which these two RBPs are attached, forming a V-shaped structure. The adaptor protein interacts with the phage virion. The V-shaped complex rotates on the adaptor – a key protein to direct the corresponding RBP downwards to the specific O-antigen degraded. Because all six V-shaped pairs in the SP6 particle mutually overlap in a so-called hand-over-hand garland, rotation of the first V-shaped pair results in the cooperative movement of all other RBP pairs. In the case of rough *Salmonella* strains lacking the long O-antigen, the V-shaped complex does not rotate; nevertheless, the phage can successfully infect these strains. It proves that rotation is not necessary to trigger the irreversible infection process in contrast to the mechanism in T4 (and suggested for G7C) [32]. In the case of the dual RBP system of phage KP32, the correct orientation of the specific RBP depending on the available capsule type will be essential as well, but the mechanism is unknown. Our data show that at least RBP1 can operate independently of RBP2.

## Conclusions

Our study provides the functional dissection of a Class 1, Group F depolymerase architecture, revealing that the CBM–LEC tandem is indispensable for proper folding, trimerization, and enzymatic activity of KP32gp38. Diverse and overlapping functions were reported for CBM and LEC domains, ranging from substrate binding over specificity determinants to condition protein trimerization, indicating the versatility of these domains amongst different RBPs. We proved that the ultimate C-terminal LEC domain is necessary and sufficient for KP32gp38 trimerization and to reconstitute its inter-subunit catalytic cleft. Trimerization, however, is not sufficient for enzymatic activity, which requires the assistance of the CBM domain for CPS recognition and digestion. Capsule binding occurs through the cooperation of all domains and the enzymatic specificity is not embedded in the CBM-LEC tandem but appears to result from the interplay of all domains. Our study and previous work showed the promiscuous and versatile nature of the CBM and LEC folds that have adopted diverse functional roles in RBPs through divergent evolution. The results obtained for the RBP analyzed here cannot be generalized to all RBPs. In some RBPs, a single domain can simultaneously function as both a trimerisation module and a determinant for enzymatic or binding activity, whereas in others these roles are carried out by distinct domains. These findings demonstrate, however, that a simple exchange of C-terminal domains is insufficient to confer or broaden host specificity, underscoring the need for a systematic approach to design engineered phages with altered specificity. To fully unravel the molecular basis of phage specificity, a comprehensive analysis of many different phage RBPs is required. Structural validation and specificity mapping in the presence of capsular polysaccharide, utilizing high-resolution methods such as X-ray crystallography or cryo-electron microscopy, could directly visualize protein structures and interactions at the level of the β-helical, CBM, and LEC domains, providing critical mechanistic insights. Additionally, molecular dynamics simulations and targeted mutagenesis would further help in identifying key determinants of binding and function. These analyses can further contribute to a more holistic understanding of phage specificity. Despite the inherent methodological challenges, including the time-intensive nature of these advanced techniques and the need for multiple iterations, we presented a robust methodological approach that allowed testing a large pool of chimeric proteins and engineered phages to demonstrate the relationships and potential functions of individual RBP domains. Together, these approaches will be highly informative for guiding the rational design of phages with tailored specificity.

## Materials and methods

### Bacterial growth conditions

*K. pneumoniae* strains were grown in Tryptone Soy Broth (TSB, Oxoid, Thermo Scientific) or on Tryptone Soy Agar (TSA; TSB supplemented with 1.5 w/v % bacteriological agar, VWR). Standard lysogeny broth (LB: 10 g Tryptone (VWR), 5 g yeast extract (VWR), 10 g sodium chloride (Fisher Scientific) or LB agar (LB supplemented with 1.5 w/v % bacteriological agar) were used to grow *Escherichia coli* strains. *E. coli* TOP10 (Invitrogen, Thermo Fisher Scientific) and *E. coli* BL21(DE3) (Invitrogen, Thermo Fisher Scientific) were used for plasmid storage and protein expression, respectively. For growth of *E. coli* transformed with a pVTE entry vector LB was supplemented with 100 μg/mL ampicillin (Carl Roth) and 5% sucrose (Fisher Scientific), while with pVTD3 or pVTD23 destination vectors, LB was supplemented with 50 μg/mL kanamycin (Carl Roth) and 5% sucrose.

### Protein production

#### Construction of truncated, chimeric and GFP-fused RBPs.

The VersaTile technique was applied to make truncated and chimeric RBPs as described previously [36,50]. First, a repository of building blocks (tiles) was created in pVTE with VersaTile cloning. Subsequently, selected tiles were combined in a single assembly reaction into the pVTD3 vector (for protein expression with C-terminal His-tag) or pVTD23 vector (for phage engineering) using VersaTile shuffling. To prepare tiles, PCR was performed using Phusion DNA polymerase (Thermo Fisher Scientific) and primer pairs (Tables F-I in S1 File), designed to flank gene of interest with position tags, which determine the position of the tile in the final assembly. Only templates that lack a SapI or BsaI recognition site were used [36]. The amplicons were purified using the GeneJET gel extraction kit (Thermo Fisher Scientific) and inserted into the pVTE entry vector by type IIs cloning (SapI/T4 DNA ligase; 15 cycles of 2 min at 37°C and 3 min at 16°C). Enzymes were inactivated by incubation at 50°C for 5 min and 65°C for 20 min. Restriction/ligation mixtures were used for the transformation of chemocompetent *E. coli* TOP10 cells by heat shock. Positive constructs were verified by colony PCR and Sanger sequencing (LGC Genomics) using appropriate primers (Table J in S1 File). Plasmid DNA of tiles was isolated from overnight cultures with the GeneJET plasmid miniprep kit (Thermo Fisher Scientific). The VersaTile shuffling reaction for assembly of the chimeric/truncated/GFP-fused variants was composed of 50 ng of each tile for respective position, 100 ng of pVTD3 destination vector, 10 U of BsaI (Thermo Fisher Scientific), 5 U of T4 DNA ligase (Thermo Fisher Scientific), 10x ligation buffer, and ultrapure water (total volume of 20 μl). Thirty cycles of restriction/ligation (2 min at 37°C and 3 min at 16°C) were followed by enzyme inactivation for 5 min at 50°C and 5 min at 80°C. Chemocompetent *E. coli* TOP10 cells were transformed with restriction/ligation mixtures by heat shock. *E. coli* BL21(DE3) cells were transformed with the correct plasmids verified by Sanger sequencing for protein expression. Site-directed mutagenesis of KP32gp38_WT was performed to create an enzymatically inactive mutant (GFP_KP32gp38_mut and GFP_KP32gp38_CP-E_mut) using KAPA HiFi polymerase (Roche) and appropriate primer pairs (Table J in S1 File) followed by DpnI (Thermo Fisher Scientific) digestion of the template. The construct composition and sequence can be found in Table K in S1 File.

#### Protein expression.

*E. coli* BL21(DE3) with the appropriate plasmids were grown in LB supplemented with 50 μg/mL kanamycin at 37°C with 180 rpm until OD_600_ reached 0.4-0.5. Expression was induced with 0.2 mM IPTG (isopropyl-β-D-thiogalactopyranoside (Carl Roth)) at 20°C for 18 h. Cultures were pelleted by centrifugation (3220 x g, 30 min, 8°C) and resuspended in lysis buffer (A: 20 mM NaH_2_PO_4_ (Fisher Scientific), 500 mM NaCl, 10 mM imidazole (Carl Roth) pH 7.4 or B: 20 mM Tris-HCl (Carl Roth), 300 mM NaCl, 5% (v/v) glycerol (Carl Roth), 10 mM imidazole (Carl Roth) pH 7.8) supplemented with DNaseI (Sigma-Aldrich; final concentration 10 µg/mL) and phenylmethylsulfonylfluorid (PMSF; Carl Roth; final concentration 1 mM). Cell lysis was done by three cycles of freeze-thawing followed by sonication. For spot tests, cell lysate was used, for other tests, proteins were purified using His GraviTrap (VWR) or HisPur Ni-NTA Superflow Agarose (Thermo Scientific).

#### Protein purification.

Lysate was clarified by centrifugation (30 min, 16,600 x g, 8°C) and filtered through a 0.2 μm syringe filter (VWR) before application on the His GraviTrap column (VWR). Columns were pre-equilibrated with lysis buffer supplemented with 10 mM imidazole. At least a 10-column volume (CV) wash was applied with the lysis buffer with 50 mM imidazole. The protein was eluted with lysis buffer with 500 mM imidazole and dialyzed against phosphate-buffered saline (PBS: 137 mM NaCl, 2.7 mM KCl (Carl Roth), 10 mM Na_2_HPO_4_, 1.8 mM KH_2_PO_4_ (Carl Roth), pH 7.4) overnight at 4°C or buffer exchange was performed using Zeba Spin Desalting Columns (Fisher Scientific). GFP-fused proteins were purified HisPur Ni-NTA Superflow Agarose (Thermo Scientific), using buffers described above. Protein fractions were analyzed via sodium dodecyl sulphate-polyacrylamide gel electrophoresis (SDS-PAGE, 180 V for approximately 1 h) in 12% polyacrylamide gels. PageRuler Unstained Broad Range Protein Ladder (Thermo Fisher Scientific) was utilized for protein molecular weight (MW) estimation. Protein visualisation was performed via Coomassie Brilliant blue G 250 (Merck) staining. Protein concentration was determined using Quick Start Bradford 1x Dye Reagent (BioRad) according to the manufacturer’s instructions.

### Phage engineering

#### Construction of chimeric RBP clusters for phage engineering.

The VersaTile technique was applied to make chimeric RBP clusters as described above, with some modifications. The VersaTile shuffling reaction was performed in two steps. In the first step, only tiles were included, while in the second step, the first assembly was inserted into the vector. The first reaction mixture was composed of 46 nM tile per position, 15 U BsaI, 3.5 U T4 DNA ligase, 10x ligation buffer, and ultrapure water (total volume of 20 μl). Thirty cycles of restriction/ligation were performed (5 min at 37°C and 5 min at 22°C). Next, 23 nM pVTD23 destination vector, 5 U BsaI, and 1.875 U T4 DNA ligase were added to the preassembled mixture and thirty additional cycles of restriction/ligation were performed (5 min at 37°C and 5 min at 22°C) followed by enzyme inactivation (5 min at 50°C and 5 min at 80 °C) and transformation of chemically competent *E. coli* TOP10. Plasmids containing the RBP clusters were extracted from overnight cultures with the GeneJET plasmid miniprep kit. The correctness of the assembly of RBP clusters was verified by colony PCR and Sanger sequencing. Primer pairs for tile creation and tile composition of the RBPs clusters are listed in Tables I and L in S1 File, respectively.

#### Phage genome engineering using Gibson assembly.

The phage genome was divided into five fragments (F1-F5) and amplified using PrimeStar (TaKaRa) polymerase or KAPA HiFi DNA polymerase (Roche) and specific primers (Table M in S1 File). Each fragment started and ended with around 200 bp overlap with two adjacent fragments, necessary for genome assembly in the Gibson reaction. Fragment four (F4) corresponded to the RBP cluster. To replace WT F4, PCR was performed on a plasmid containing an assembled engineered RBP cluster (eF4) for linearization. A Gibson assembly mixture contained 0.05 pM of each linear fragment, purified using DNA Clean & Concentrator kit (ZYMO RESEARCH), and Gibson master mix ([51]; Table N in S1 File). Gibson assembly was performed for 30 min at 37°C, followed by 60 min at 60°C and enzyme inactivation for 20 min at 80°C. Afterward, the mixture was dialysed against ultrapure water using MF-Millipore 0.025 µm MCE Membrane (Fisher Scientific) and used for electroporation of electrocompetent *E. coli* 10G ELITE cells (LGC Genomics). The electroporated cells were restored in the SOC medium (LGC Genomics) and incubated for 3h at 37°C with shaking. After that, 1:10 chloroform was added to release the rebooted, engineered virions, and the mixture was vortexed and centrifuged (5 min, 1700 x g). The supernatant was plated on a *K. pneumoniae* 271 lawn, using the double agar overlay method [52]. After overnight incubation, a single plaque was selected per engineered phage, the phage was propagated using liquid propagation (see below), the phage genome was extracted using a Quick-DNA Viral kit (ZYMO RESEARCH), and its correctness (compared to the design) was verified with Nanopore sequencing (Plasmidsaurus).

### Protein and phage assays

#### Activity/specificity spot test.

Bacterial lawns for protein activity/specificity tests were prepared by pouring 1 mL of grown *Klebsiella* culture (OD_600_= at least 1 but adjusted to OD_600_ = 1 when MHC was tested) on TSA plates, distributed, and left to dry. Ten microliters of lysate (total fraction after sonication) or purified (and serially diluted) protein were spotted on the dried bacterial lawn with 10 μL lysis buffer, PBS, and BL21(DE3) lysate (with an empty plasmid) as controls. Plates were incubated at 37°C for 18 h and checked for halo formation. The lowest concentration with a visible halo was defined as the minimum halo-formation concentration (MHC). The screening of the entire *Klebsiella* capsular type collection was done once. All other experiments on smaller strain subsets were performed in triplicate.

#### GFP-based binding assay.

A previously reported fluorescence-based binding assay [53] with some modifications was used. *K. pneumoniae* strains (in triplicate) were grown in TSB to at least OD_600_ = 1. Cultures were centrifuged (5 min, 1700 x g), the supernatant was discarded and the pellet was washed with PBS twice. The washed pellet was resuspended in PBS to a final OD_600_ = 1. To 96-well fluorescence plates (Nunclon 96 Flat Bottom Black Polystyrene, Thermo Fisher Scientific) 180 µL of each bacterial suspension at OD_600_ = 1 was transferred, and 20 µL of respective GFP constructs, adjusted with PBS to 10 µM concentration (1 µM final protein concentration), were added. Bacteria mixed with respective proteins were incubated for 10 min at room temperature, in the dark. After that, the plate was centrifuged at 3220 x g for 5 min and the supernatant was discarded. Bacteria-protein pellets were washed once by adding 200 µL of PBS and pipetting up and down, followed by centrifugation and removal of the supernatant. Washed pellets were resuspended in 200 µL of PBS. The fluorescence was measured (excitation 485 nm, emission 530 nm, orbital shaking 3 mm amplitude for 6 s) with Infinite 200 PRO (Tecan). Each condition was performed in triplicate. Results were presented as relative fluorescence units (RFU): the blank was subtracted from the fluorescence measurements and the results were divided by the mean of the values of the positive control.

#### Decapsulation of Kp968 (KL163) for binding assay.

Bacterial cells were grown and washed with PBS as described above, and the culture was adjusted to an OD₆₀₀ = 1. Decapsulation was performed by adding depolymerase KP32gp38 to a final concentration of 1 µM, followed by incubation for 1 h at 37 °C. After incubation, the cells were pelleted by centrifugation (5 min, 1700 x g) and washed once with PBS. The washed pellet was then resuspended in PBS and adjusted again to an OD₆₀₀ = 1. This decapsulated cell suspension was used in the binding assay as described above.

#### Phage propagation.

The respective *K. pneumoniae* hosts were inoculated into TSB and grown at 37°C with shaking. Three rounds of phage propagation were performed, where in each step the phage suspension was diluted ten times with the respective bacterial cultures (host for RBP1 and host for RBP2). A bacterial culture at OD_600_ ~ 0.1/0.2 was mixed with phage suspension in a 9:1 ratio. After 1h incubation at 37°C with shaking, 100 µL of chloroform was added, vortexed, and centrifuged (1700 x g, 5 min, 4°C). Supernatant was collected and used for the second round of propagation as described above. Supernatant from the second round of propagation was used for the third round of propagation. The titer of the starting phage suspension, expressed as log10 (PFU/mL) as well as the titer of suspensions after three rounds of propagation on two phage hosts (host for RBP1 and RBP2) were determined using a spot test. First, bacterial lawns (in triplicate) were prepared using *K. pneumoniae* cultures at OD_600_= at least 1. To 4 mL of the soft agar (0.5 w/v % agar in TSB) 200 µL of the respective bacterial culture (phage host for RBP1 or RBP2) was added and poured on a TSA plate. After solidifying and drying, the lawns were used to spot phage suspension. For each phage suspension, three 10-fold dilution series were prepared in PBS and 10 µL of each dilution was spotted on the bacterial lawns. After overnight incubation at 37°C, plaques were counted and the titer was calculated. The starting titer divided by 1000 (the phage suspension was diluted 10 times in each of the three consecutive rounds of propagation) was subtracted from the titer achieved after propagation to determine effective propagation expressed as Δlog10 (PFU/mL).

### Statistical analysis

The Shapiro-Wilk test was run to check the normality and lognormality of data, followed by the creation of a QQ plot. Ordinary one-way ANOVA followed by Tukey’s multiple comparisons tests (GraphPad Prism 9, San Diego, CA, USA) were run to test if there was a statistically significant difference. Stars indicate statistically significant differences: * for p < 0.05; ** for 0.01 < p < 0.05; *** for 0.0001 < p < 0.01; **** for p < 0.0001.

## Supporting information

S1 File Table A. Minimal halo-forming concentration (MHC) of truncated variant compared to wild-type KP32gp38. Table B. Enzymatic activity of truncated variants and individual domain(s) of RBP2 (KP32gp38) screened on *Klebsiella* capsular type collection. Table C. Enzymatic activity of chimeric variants obtained by the fusion of KP32gp38 C-terminal domains with other RBPs with depolymerase activity. Table D. Enzymatic activity of chimeric variants obtained by the fusion of KP32gp38 C-terminal domains with other RBPs with depolymerase activity screened on *Klebsiella* capsular type collection. Table E. Enzymatic activity of controls of chimeric variants obtained by the reassembly of KP32gp38 and K11gp0043 with position marker (PM) between domains. Table F. Primers used for tile creation of truncated RBPs, together with the tile sequence. Table G. Primers used for tile creation of chimeric RBPs, together with the tile sequence. Table H. Primers used for tile creation of (truncated) RBP fusions with GFP, together with the tile sequence. Table I. Primers used for tile creation to assemble RBP gene clusters of engineered phages (ePs), together with the tile sequence. Table J. Primers for colony PCR, Sanger sequencing and site-directed mutagenesis. Table K. Tile composition of truncated, chimeric and GFP-fused RBPs together with total construct sequence. Table L. The composition of tiles to assemble RBPs gene cluster of engineered phages (ePs). Table M. Primers used for amplification of the KP32 genome fragments (F1-F5). Table N. Components for the preparation of 5X ISO buffer and the Gibson master mix (51) for the assembly of the genomes of the engineered phages. Fig A. Three‑dimensional structures of KP32gp38, its truncated variants, and individual domains predicted using AlphaFold 3 (27). Fig B. Three‑dimensional structures of the wild‑type RBPs used in this study, predicted using AlphaFold 3 (27). Fig C. Three‑dimensional structures of the chimeric RBPs predicted using AlphaFold 3 (27). Fig D. Three‑dimensional structures of the chimeric RBPs (between KP32gp38 and K11gp0043) predicted using AlphaFold 3 (27). Fig E. Fluorescence-based binding assay. Fig F. Three‑dimensional structures GFP-fusions of KP32gp38, its truncated variants, and individual domains predicted using AlphaFold 3 as trimers (27). Fig G. Three‑dimensional structures of KP32gp38, its truncated variants, and individual domains and their GFP-fusions predicted using AlphaFold 3 as monomers (27). Fig H. Graphical abstract.
(XLSX)

S1 Fig (TIF)

S2 Fig (TIF)
