# Supplementary figures and images for: Role of the C-terminal modules of Klebsiella phage KP32 receptor-binding protein gp38 in protein and phage functionality
Source: PLoS Pathog. 2026 Apr 6;22(4):e1014106. doi: 10.1371/journal.ppat.1014106 (PMC13082707; doi:10.1371/journal.ppat.1014106)

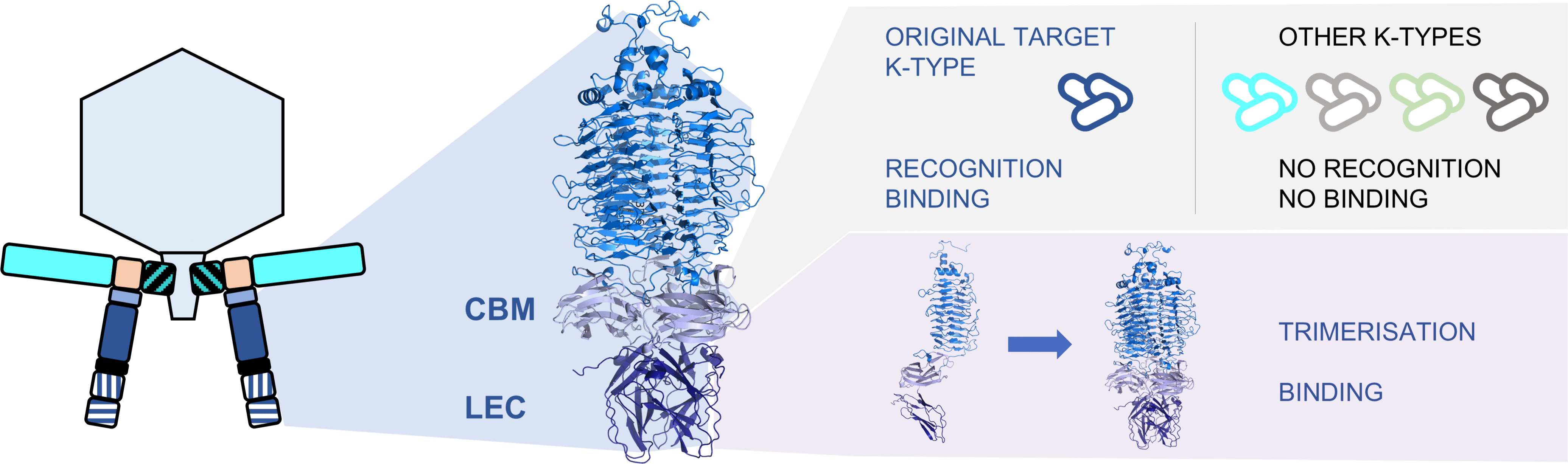

Supplement: S1 Fig — (TIF) [file ppat.1014106.s002.tif]

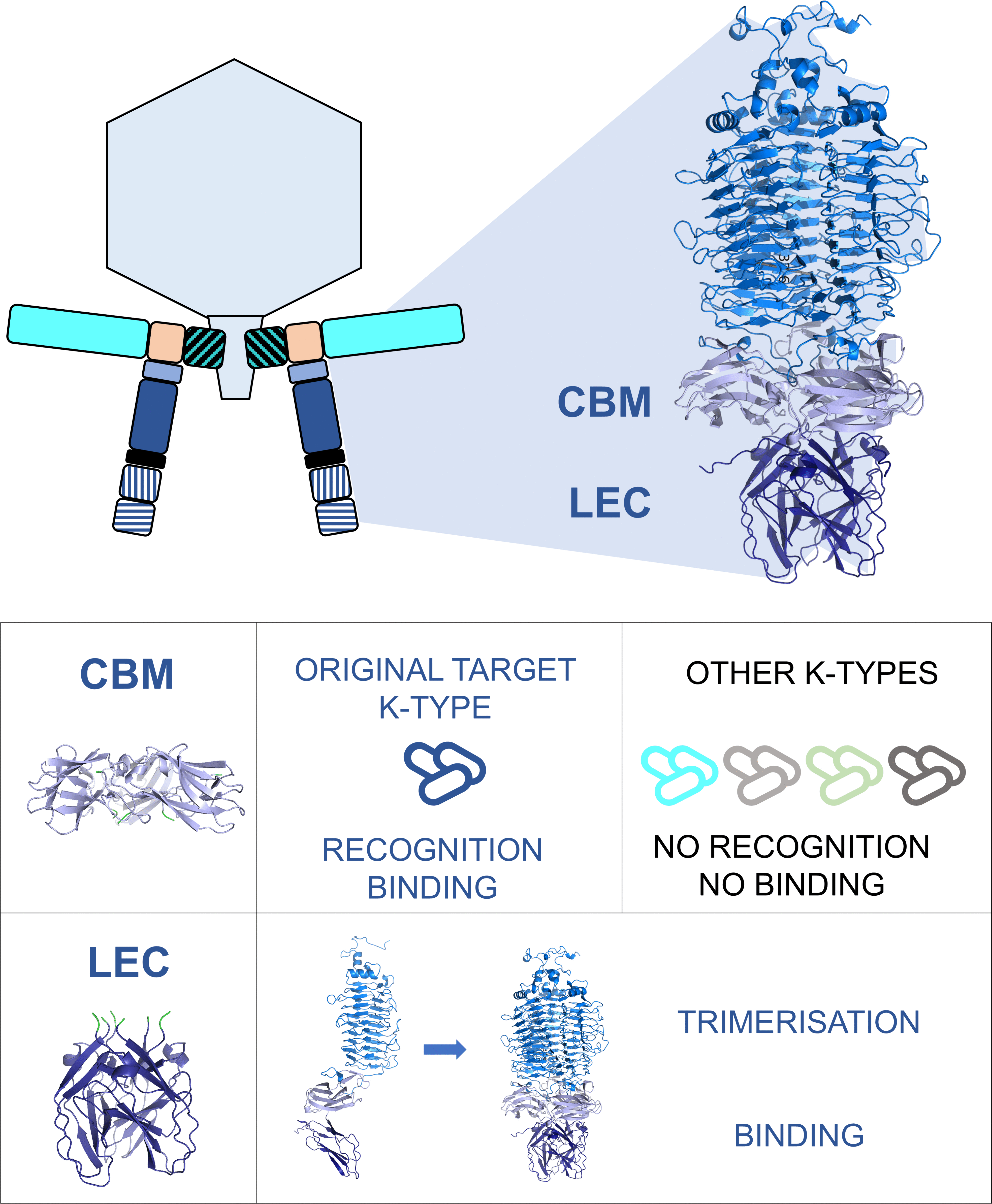

Supplement: S2 Fig — (TIF) [file ppat.1014106.s003.tif]
